# ExpoKids: An R-based tool for characterizing aggregate chemical exposure during childhood

**DOI:** 10.1038/s41370-020-00265-6

**Published:** 2020-10-05

**Authors:** Mona Dai, Susan Y. Euling, Linda Phillips, Glenn E. Rice

**Affiliations:** 1 Oak Ridge Institute for Science and Education at US EPA, Office of Children’s Health Protection (OCHP), Washington, DC USA; 2 US EPA, OCHP, Washington, DC USA; 3 US EPA, National Center for Environmental Assessment, Washington, DC USA; 4 US EPA, Center for Public Health and Environmental Assessment, Chemical & Pollutant Assessment Division, Cincinnati, OH USA; 5 Present Address: Harvard John A. Paulson School of Engineering and Applied Sciences, Cambridge, MA USA

## Abstract

**Background:**

Aggregate exposure, the combined exposures to a single chemical from all pathways, is a critical children’s health issue.

**Objective:**

The primary objective is to develop a tool to illustrate potential differences in aggregate exposure at various childhood lifestages and the adult lifestage.

**Methods:**

We developed ExpoKids (an R-based tool) using oral exposure estimates across lifestages generated by US EPA’s Exposure Factors Interactive Resource for Scenarios Tool (ExpoFIRST).

**Results:**

ExpoKids is applied to illustrate aggregate oral exposure, for ten media, as average daily doses (ADD) and lifetime average daily doses (LADD) in five graphs organized across seven postnatal childhood lifestages and the adult lifestage. This data visualization tool conveys ExpoFIRST findings, from available exposure data, to highlight the relative contributions of media and lifestages to chemical exposure. To evaluate the effectiveness of ExpoKids, three chemical case examples (di[2-ethylhexyl] phthalate [DEHP], manganese, and endosulfan) were explored. Data available from the published literature and databases for each case example were used to explore research questions regarding media and lifestage contributions to aggregate exposure.

**Significance:**

These illustrative case examples demonstrate ExpoKids’ versatile application to explore a diverse set of children’s health risk assessment and management questions by visually depicting specific media and lifestage contributions to aggregate exposure.

## Introduction

Aggregate exposure assessments evaluate the combined exposures to a single chemical across multiple routes (oral, dermal, inhalation) and multiple exposure media/pathways (food, drinking water, dust, etc.). Aggregate exposure assessments are often important in children’s health risk assessments because children’s unique behaviors and physiology can alter chemical exposure rates across different media, routes, and lifestages relative to the adult lifestage. Further, relative to adults, children exhibit increased susceptibility to some chemical exposures during development, defined as a series of temporally and spatially orchestrated events from zygote implantation to completion of puberty [1–3]. Generally, chemical intake rates may be higher per unit body weight for children than for adults [2].

Aggregate exposures are addressed differently among and within organizations. The Food Quality Protection Act (FQPA) requires the US Environmental Protection Agency (EPA) to “ensure… reasonable certainty that no harm will result to infants and children from aggregate exposure to the pesticide chemical residue” [4]. In response, EPA published guidance for evaluating pesticide aggregate exposures [5]. Similarly, amendments to the Toxic Substances Control Act require EPA to “describe whether aggregate or sentinel exposures to a chemical substance [are] considered” as part of risk assessment [6]. Other federal organizations have developed similar methods for assessing aggregate exposure [7]. Outside of the USA, aggregate exposure assessment approaches for evaluating pesticide and consumer product exposures have also been created [8–10]. While efforts to comprehensively consider aggregate exposures from all pathways and routes have been developed, implementation has been limited by data availability for individual chemicals.

We developed ExpoKids to visualize contributions of multiple oral media to aggregate exposures both within and across lifestages. To our knowledge, the development of such approaches has been limited. This tool can effectively visually communicate and compare aggregate exposure information using available exposure data. Although ExpoKids can be used independently, it was developed to work with the Exposure Factors Interactive Resource for Scenarios Tool (ExpoFIRST; https://cfpub.epa.gov/ncea/efp/recordisplay.cfm?deid=344928). We first describe the development of ExpoKids. Next, we explore the use of ExpoKids to visually illustrate the contributions of ten different oral media to seven lifestage-specific aggregate exposure estimates displaying average daily dose (ADD) and lifetime average daily dose (LADD). ADD refers to the dose rate averaged over a specified exposure interval and expressed as a daily dose on a per unit body weight basis. LADD describes the dose rate averaged over an individual’s anticipated lifetime. The effectiveness of ExpoKids is then evaluated by posing three routine exposure assessment questions that highlight the potential utility of ExpoKids for a variety of stakeholders interested in children’s environmental exposures.

## Methods

ExpoKids Version 1.0 was developed in R (Version 3.4.0) and can be used as an extension of ExpoFIRST to illustrate aggregate exposure estimates. ExpoFIRST is a standalone tool that utilizes the EPA’s *Exposure Factors Handbook (EFH): 2011 Edition* to provide deterministic potential dose estimates for user-defined exposure scenarios [2]. The EFH summarizes available human exposure data, providing exposure factor estimates [2].

### ExpoFIRST Integration

Using ExpoFIRST (Version 2.0), we estimated the ADDs from the intake of ten media (i.e., soil, dust, water, breastmilk, dairy, meat, fish, vegetables, fruit, and grains) [2]. ADD was chosen as the ExpoKids metric to capture typical exposures experienced by average Americans. ExpoFIRST itself did not evaluate aggregate exposure, but rather ran ADD estimates for each medium separately. Subsequently, we exported the medium-specific ADD estimates from ExpoFIRST into ExpoKids to develop aggregate exposure graphs. Central tendency oral ADDs (mg/kg-day) for the EFH’s ten children’s age groups and the adult age group (Table 1) within the general population were estimated in ExpoFIRST for each medium using the following general equation [2]:

$${\rm{ADD}} = \frac{{C \times {\rm{IR}} \times {\rm{EF}} \times {\rm{ED}}}}{{{\rm{AT}} \times {\rm{BW}}}}$$

**Table 1:** ExpoKids recategorizes the *Exposure Factors Handbook* (*EFH*) age groups into lifestages based on the EPA’s *Guidance on Selecting Age Groups for Monitoring and Assessing Childhood Exposures to Environmental Contaminants*.

	EFH age groups	ExpoKids lifestage	Total years in lifestage
Childhood	Birth to <1 month	Young Infant^a^	1
	1 to <3 months		
	3 to <6 months		
	6 to <12 months		
	1 to <2 years	Infant^a^	2
	2 to <3 years		
	3 to <6 years	Young child	3
	6 to <11 years	Child	5
	11 to <16 years	Young youth	5
	16 to <21 years	Youth	5
	21 to <70 years	Adult	49
	Birth to <70 years	Lifetime	70

^a^The “young infant” and “infant” lifestages are the only ExpoKids lifestages to combine multiple EFH/ExpoFIRST age groups.

Above, *C* = concentration (mg/mL or mg/g), IR = intake rate (mL/kg-day, g/kg-day, or mg/day), EF = exposure frequency (days/year), ED = exposure duration (years), AT = averaging time (days), and BW = body weight (kg) [2]. Age-specific central tendency estimates (either mean or median, depending on the exposure factor) from the EFH were used for IR, EF, ED, AT, and BW [2]. Chemical concentrations (*C*) were based on media-specific values from data identified in the scientific literature. ExpoFIRST allows users to define an unlimited number of potential scenarios for various populations and lifestages. In running ExpoFIRST for our illustrative case examples, we selected parameters representing general population exposure.

While we recognize that our assumptions have some limitations, they were selected to be consistent and compatible with ExpoFIRST outputs. For the case examples, we estimated average wet weight concentrations based on data representative of an entire medium, even if only a subset was sampled. For all food categories, we estimated exposure for “total” food groups (e.g., total fruits, total vegetables), developing “per capita” estimates for males and females combined. For drinking water, we estimated exposure to chemicals in community water sources, thereby assuming that contaminants were only present in tap water (i.e., not bottled water) to be consistent with general population exposure scenarios. Once ADDs were estimated from ExpoFIRST, chemical-specific tables were exported and reorganized into Excel tables displaying ADDs for all media by lifestage and then input into ExpoKids to create graphs (see user guide [S-1: Fig. S1, Tables S1–4] in Supplementary Information for details).

For children under 1 month of age, EFH values were unavailable for soil and dust ingestion; both intake rates were assumed to be 0 mg/day. Furthermore, due to a lack of available data on formula-fed infants, only breastfeeding data for young infants was included in ExpoFIRST v2.0.

### ExpoKids development

ExpoKids can create five unique graphical displays of ADD by lifestage, LADD by lifestage, percent ADD by lifestage, ADD for individual exposure pathways, and LADD for individual exposure pathways (Table 2). Since the EFH followed the EPA’s *Guidance on Selecting Age Groups for Monitoring and Assessing Childhood Exposures to Environmental Contaminants* as available data allowed, ExpoKids followed a similar renaming structure for reorganizing age groups into lifestages [1]. The ingestion pathway, for instance, did not have data for all age groups under 1 year of age and the EFH collapsed these four age groups into one, named young infants in ExpoKids. The infant lifestage in ExpoKids treated the 1–3-year-old EFH age groups similarly. Thus, eleven ExpoFIRST/EFH age groups were converted to seven ExpoKids lifestages (Table 1). This facilitated comparisons among six childhood lifestages, as well as between childhood in total (birth to less than 21 years of age), adulthood (21 to less than 70 years of age), and lifetime (birth to less than 70 years of age) ADD. After the data tables were uploaded (R package: readxl), the *melt* function (R package: reshape2) rearranged the data into a readable format for the statistical program to create stacked bar plots using the *ggplot* function (R package: tidyverse). The resulting eleven graphs (one all media graph and ten medium-specific graphs) displayed the estimated ADD values by lifestage. LADD values were then estimated by time-weighting each ADD value; i.e., each ADD was multiplied by a ratio of time spent within each lifestage divided by the total lifespan (70 years). LADDs were calculated in ExpoKids to streamline calculations and prevent the need for multiple data exportations into ExpoKids. Estimates were performed and verified by the first author (MD).

**Table 2:** Five lifestage and oral media data visualization graph types produced by ExpoKids: ADD by lifestage, LADD by lifestage, percent ADD by lifestage, ADD for an individual exposure pathway, and LADD for an individual exposure pathway.

Graph type	Example graph
1. ADD by lifestage: compares lifestages and media as relative contributors to an individual’s lifetime exposure.	
2. LADD by lifestage: compares lifestage and media ADD contributions scaled by the number of years an individual spends in each lifestage (time duration within a lifestage is considered).	
3. Percent ADD by lifestage: calculates relative ADD contributions within a lifestage as relative percentages within a lifestage.	
4. ADD for an individual exposure pathway: focuses on illustrating the relative contribution between lifestages for a specific medium of interest (10 total).	
5. LADD for an individual exposure pathway: focuses on illustrating the relative contribution between lifestages for a specific medium of interest (10 total) scaled by the number of years an individual spends in each lifestage.	

For each medium, ADD per lifestage of interest (ADD_*j*_) was estimated from the age groups using the following equation:

$${\rm{ADD}}_j = \frac{{{\sum} {({\rm{ADD}}_i \times Y_i)} }}{{{\sum} {Y_i} }}$$

in which ADD_*i*_ is the ADD value from the ExpoFIRST age group within the relabeled lifestage of interest, *Y*_i_ is the length in years of that age group, ∑*Y*_i_ is the total number of years in the new lifestage, and i represents the age group within the lifestage being estimated.

LADD per lifestage (LADD_*j*_) of interest for the media was estimated from:

$${\rm{LADD}}_j = \frac{{{\rm{ADD}}_j \times Y_j}}{{{\rm{Lifespan}}}}$$

ADDs were also converted to percentages to estimate the percent contribution of each medium within a lifestage using the following equation:

$${\mathrm{\% }}\,{\rm{Lifestage}}\,{\rm{contribution}}_j = \frac{{\rm{ADD}}_j}{{{\sum} {{\rm{ADD}}_j} }} \times 100{\mathrm{\% }}$$

### Case example data selection

To evaluate the effectiveness of ExpoKids, we developed illustrative exposure scenarios involving environmental chemicals and explored three questions that often arise in exposure assessments:

1. What are the relative contributions of specific media across lifestages to lifetime aggregate chemical exposure?
2. Are there differences in exposures across lifestages for an essential nutrient that is associated with developmental toxicity at elevated exposure rates?
3. How has an environmental regulation affected aggregate exposure rates to a chemical across lifestages?

Our criteria for selecting environmental chemicals to explore the above research questions included: (1) evidence of developmental toxicity after exposure; (2) evidence of at least one critical or sensitive window of exposure during postnatal development; and (3) available chemical-specific concentration data in at least four oral exposure media included in ExpoFIRST. Following general assessment procedures to identify appropriate data [11], we searched PubMed in September 2018 to identify peer reviewed articles with clear descriptions of their scientific methodology (see S-3: Table S1 for search terms). Next, we scanned the article titles and abstracts to ensure that the contents were useful for our illustrative case examples. We then applied the following inclusion criteria to the studies:

1. Published between January 2000 and September 2018.
2. Collected data in the USA, preferably nationwide.
3. Quantitatively measured chemical levels directly in media.
4. Conducted from a general population perspective.

All articles retrieved were reviewed to ensure that the general analysis, assumptions, and data were complete, and that variability was evaluated and characterized. To gauge the accuracy of the selected chemical concentrations, an additional literature search was conducted on the largest relative ADD medium for each chemical (see S-3: Tables S2–4). Within each search, all relevant articles were collected for each chemical to compare their mean concentration inputs.

We next searched for candidate chemical concentration data. Our inclusion criteria included regularly maintained databases that used well-established methods with at least five years of monitored and/or measured US data. We also performed literature searches using keywords for the chemical of interest in each of the ten media to identify articles reporting media-specific concentrations. Mean media concentrations for chemicals were selected from a single data source keeping with the following hierarchy—We preferred mean background concentration measures from recently published US nationwide databases and excluded measures from sampling locations with known elevated chemical concentrations (such as near chemical spills or industrial sites). Only measured chemical-specific concentrations were collected for each case example. Therefore, the accuracy of chemical-specific concentrations relied heavily on measurement methods rather than models. Chemical-specific concentrations identified in the USA were prioritized over international chemical concentrations to avoid capturing differences in chemical production, use, or regulations from other countries. If an appropriate US database could not be found, we used the most recently published national study providing chemical concentrations measured in the media of interest. If nationwide data were not available, regional US studies were sought. When raw measurement data were collected, the arithmetic mean concentration was calculated for each medium. We also collected information regarding limit of detection (LOD), percent of samples above LOD, total number of samples measured (*n*), median, and standard deviation (SD) (Tables 3–5). Since only one study or data source was chosen per medium, aggregate statistics were not calculated. Measurements below the LOD were excluded from mean estimates unless otherwise specified for the purposes of the illustrative case examples. Other non-detect approaches may be appropriate as well.

**Table 3:** Statistical descriptions of measured DEHP concentrations in media for the illustrative case example.

Media	*n*	Samples > LOD	LOD	Mean	Median	SD	Reference
Dust (mg/g)	11	100%	0.14–278 ng/mL [dust/hexane: acetone]	9.73E−02	7.31E−02	1.11E−01	Subedi et al. [16]
Soil (mg/g)	6	100%	0.2 µg/mL 1-butanol buffer	9.39E−03	8.63E−03	2.19E−03	Lin et al. [17]
Water (mg/mL)	15	13%	1.76E-06	2.56E−06	N/A	N/A	Loraine et al. [18]
Breastmilk (mg/mL)	21	100%	MEHP = 6.18E−7 MEOHP = 1.03E−7 MEHHP = 1.03E−7	7.07E−04	1.91E−04	8.87E−04	Hartle et al. [19]
Dairy (mg/g)	11	100%	3.70E−06	1.26E−04	6.97E−05	1.25E−04	Schecter et al. [20]
Meat (mg/g)	13	69%	3.70E−06	1.01E−04	7.00E−06	3.18E−04	Schecter et al. [20]
Fish (mg/g)	5	80%	3.70E−06	3.14E−05	3.96E−05	2.60E−05	Schecter et al. [20]
Vegetables (mg/g)	5	40%	3.70E−06	5.09E−06	1.85E−06	8.70E−06	Schecter et al. [20]
Fruit (mg/g)	5	40%	3.70E−06	5.09E−06	1.85E−06	8.70E−06	Schecter et al. [20]
Grains (mg/g)	7	100%	3.70E−06	6.16E−05	5.06E−05	4.25E−05	Schecter et al. [20]

**Table 4:** Statistical descriptions of measured manganese concentrations in media for the illustrative case example.

Media	*n*	Samples > LOD	LOD	Mean	Median	SD	Reference
Dust (mg/g)	661	N/A	N/A	2.22E−01	1.60E–01	2.45E−01	Gulson et al. [31]
Soil (mg/g)	329	N/A	N/A	3.41E−01	1.80E–01	1.90E−01	Gulson et al. [31]
Water (mg/mL)	416	57%	9.00E−07	6.35E−05	2.80E–06	2.19E−04	Lindsey et al. [33]
Breastmilk (mg/mL)	20	N/A	N/A	2.71E−06	N/A	1.12E−06	Klein et al. [32]
Dairy (mg/g)	759	25%	3.0E−4 to 4.0E−4	8.19E−04	1.30E–04	1.46E−03	TDS 2006–2013 [34]
Meat (mg/g)	1725	65%	3.0E−4 to 4.0E−4	1.66E−03	1.70E–03	1.06E−03	TDS 2006–2013 [34]
Fish (mg/g)	224	46%	3.0E−03	9.63E−04	4.20E–04	1.10E−03	TDS 2006–2013 [34]
Vegetables (mg/g)	1631	99%	2.0E−4 to 4.0E−4	1.79E−03	1.20E–03	1.31E−03	TDS 2006–2013 [34]
Fruit (mg/g)	1055	79%	2.0E−4 to 4.0E−4	1.62E−03	4.45E–04	3.45E−03	TDS 2006–2013 [34]
Grains (mg/g)	1484	99%	3.0E−4 to 4.0E−4	7.34E−03	4.90E–03	7.14E−03	TDS 2006–2013 [34]

**Table 5:** Statistical descriptions of measured endosulfan sulfate concentrations (positive detects only) for the illustrative case example from USDA’s Pesticide Database Program (PDP) [44].

Media	*n*	Samples > LOD	LOD	Mean	Median	SD
Pre Phase-Out: 1994–2010						
Dairy (mg/g)	5220	14.80%	3.00E−08 to 1.00E−06	2.55E−06	2.00E−06	7.45E−03
Meat (mg/g)	1176	1.80%	3.00E−07 to 2.00E−06	1.48E−05	4.80E−06	2.67E−02
Fish (mg/g)	1479	6.60%	1.00E−06	5.26E−06	2.70E−06	6.03E−03
Vegetables (mg/g)	61805	11.70%	2.00E−06 to 4E−04	3.80E−05	2.00E−05	7.49E−02
Fruit (mg/g)	39871	4.60%	1.00E−06 to 6.80E−02	2.16E−05	1.20E−05	2.63E−02
Grains (mg/g)	558	0.20%	1.00E−06	2.00E−06	2.00E−06	0.00E+00
Post Phase-Out: 2011–2016						
Vegetables (mg/g)	21355	4.40%	2.00E−06 to 4.00E−04	2.53E−05	1.40E−05	3.49E−05
Fruit (mg/g)	7415	1.50%	1.00E−06 to 6.80E−02	2.26E−05	1.90E−05	1.28E−05

## Results

### ExpoKids graphs

ExpoKids visually conveys ADD and LADD findings to highlight the relative contributions of media and lifestages for environmental chemicals. ExpoKids’ five aggregate exposure graph types illustrate differences across lifestages and media (Table 2). The aggregate ADD by lifestage graph illustrates aggregate media ingestion by lifestage to demonstrate the relative contribution of each medium within each lifestage. Likewise, the aggregate graph for LADD by lifestage depicts LADD contributions scaled by time spent in each lifestage. LADD graphs are useful for visualizing the relative average daily dose of each lifestage. For instance, although an aggregate ADD graph may illustrate higher exposure rates in younger childhood lifestages, the total years in childhood lifestages account for a shorter period of time compared to adulthood. As a result, time duration within a lifestage is considered when comparing estimated LADDs. The percent ADD graph displays ADD values on a cumulative percentage scale to compare relative media contributions within lifestages. The ADD and percent ADD graphs are also generated for childhood, adult, and lifetime exposures to facilitate comparisons. Lastly, these graphs are further individualized into ten ADD and LADD graphs per exposure pathway (20 total) that focus attention on specific media of interest.

### Evaluating the effectiveness of ExpoKids

Exposure assessment questions highlight the breadth of possibilities that may be examined by ExpoKids and emphasize the importance of aggregate exposure assessments when evaluating children’s health. The results presented in the following case examples are for illustrative purposes only; alternate concentration estimates may produce different results. The estimated ADD values can be found in S-3: Tables S5–7.

#### Question 1: What are the relative contributions of specific media across lifestages to lifetime aggregate chemical exposure?

Di[2-ethylhexyl] phthalate (DEHP) was selected to illustrate this first question because of the chemical’s developmental toxicity and historic uses [12]. DEHP, a “developmentally toxic phthalate”, is associated with adverse reproductive effects following exposures during development [13]. DEHP assessments for various exposure sources (i.e., food, water, and commercial products) have been conducted by multiple groups [12–15]. Based on rat studies in which male reproductive effects were observed following gestational exposures, critical windows of exposure for phthalates and male reproductive developmental effects have been identified ranging from the most sensitive gestational exposure window, the moderately sensitive postnatal window, and the least sensitive adulthood window [13].

DEHP concentrations were extracted from five studies for the ten media of interest (Table 3). Five individual PubMed searches relating phthalates and the respective media of interest were conducted, retrieving 650 total articles. All extracted values were from regional US studies, except for soil concentration. The average DEHP concentration in dust was estimated based on phthalate dust samples collected in eleven homes from five geographically diverse US states in 2016 [16]. Since US DEHP soil data could not be identified, data collected from three fields in China over an unspecified time period were used [17]. Loraine et al. [18] reported DEHP concentrations in finished (treated) drinking water samples collected from California water treatment plants from 2001 to 2002. Hartle et al. [19] analyzed 21 human milk samples from 2015 in California; samples below the LOD were input as ${\rm{LOD}}/\sqrt 2$ . Unlike the other studies, three DEHP metabolites (MEHP, MEOHP, and MEHHP) were measured in breastmilk as a proxy for DEHP [19]. DEHP concentration in breastmilk was estimated by summing these average metabolite levels [19]. All food (dairy, meat, fish, vegetables, fruit, and grains) concentrations were extracted from Schecter et al. [20]. While relying on one study for all food groups provided consistency in data collection methodology, Schecter et al. sampled few food items overall (*n* = 41) from one state (New York) and fruits and vegetables were classified together [20].

**Fig. 1:**
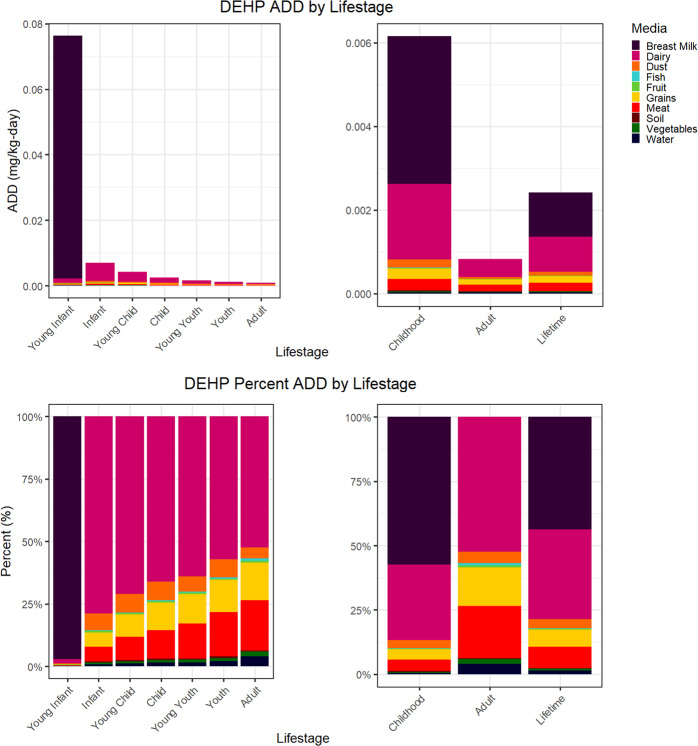
DEHP illustrative case example graphs: ADD by lifestage graphs are shown in the top row and cumulative percentage ADD by lifestage graphs are shown in the bottom row.

The DEHP ExpoKids graphs (Fig. 1) show breastmilk consumption to be the dominant DEHP exposure medium in this illustrative case example based on an intake rate per kilogram body weight basis; among the included media, over 90% of the predicted DEHP exposure was from breastmilk intake for young infants. Young infants had the highest exposure rates to DEHP among all lifestages in this case study. After this lifestage, infants had the next highest aggregate ADD estimate, with declines in ADD in subsequent lifestages. Among all media evaluated, dairy intake accounted for over 50% of estimated DEHP exposures across all lifestages except for young infants. In general, the relative contribution of all media within each lifestage remained relatively constant for individuals older than 1 year of age. Overall, during childhood, both breastmilk and dairy contributed more to ADD than other media evaluated.

#### Question 2: Are there differences in exposures across lifestages for an essential nutrient that is associated with developmental toxicity at elevated exposure rates?

Manganese was chosen as an illustrative case example for this question. The National Academies of Sciences (NAS) set a specified range for manganese between the lower limit or age-dependent adequate intake (AI) value and the tolerable upper limit (UL) value for discrete age groups to address low dose nutritional benefits and high dose neurotoxicity [21, 22]. Food is a major source of manganese intake for humans; as a result, assessing average dietary intake is important [22]. Laboratory animal studies with manganese oral doses during either gestational only, postnatal only, or gestational and postnatal developmental exposure windows reported developmental (e.g., skeletal development and growth) and neurodevelopmental toxicities [23–25]. These studies indicated that both the perinatal and the postnatal periods are critical windows of exposure. Epidemiologic studies also reported an increase in neurodevelopmental effects associated with increased prenatal or early postnatal manganese exposure [26, 27]. The EPAʼs Integrated Risk Information System (IRIS) RfD for manganese is 0.14 mg/kg-day, based on central nervous system effects and is consistent with the UL value reported by NAS based on its review of the dietary literature [21, 22]. Other agencies have also conducted health assessments of orally ingested manganese [28–30].

Table 4 lists average manganese concentration inputs to ExpoFIRST. Two studies on dust, soil, and breastmilk were identified from two PubMed searches that returned 464 articles. Since no US studies were available, dust and soil manganese concentration values were extracted from Gulson et al. [31] who sampled dust and soil collected from 108 households in Australia between 2001 and 2006. Mean manganese concentrations in breastmilk were estimated by Klein et al. [32] based on twenty 2013 breastmilk samples collected in Massachusetts. Mean manganese concentrations from 416 water samples from the United States Geological Survey’s (USGS) National Water Quality Assessment’s (NAWQA) dataset from 2013 to 2014 were also identified [33]. Since NAWQA reported median instead of mean manganese concentrations, these estimates were less outlier influenced [33]. USGS assigned half of the non-detect concentrations to be 0.05 mg/L (the LOD value) and the other half to be 0.025 mg/L (half of the LOD value) [33]. While this method may have overestimated manganese concentrations in water if the true concentrations were zero, this was unlikely since manganese naturally occurs in the environment [21]. The most recent FDA Total Diet Study (TDS) data collected from 2006 to 2013 were downloaded for food name, number of detects, LOD, and other summary statistics [34]. Each food commodity was sorted into its appropriate category based on its FDA categorization in the TDS food/analyte matrix before the mean manganese concentration for each food group was estimated [34]. All food commodities were assigned to a food category and average concentrations within each medium were estimated.

**Fig. 2:**
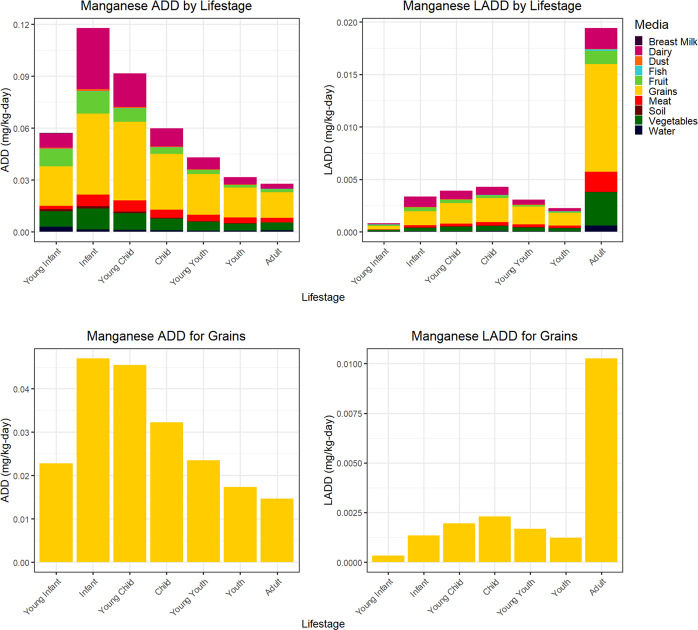
Manganese illustrative case example graphs: ADD by lifestage graphs are shown in the top row and LADD by lifestage for exposure to grains is shown in the bottom row.

Figure 2 presents aggregate ADD manganese ExpoKids graphs based on inputs from Table 4. Manganese intake rates for all lifestages were between the AI and the UL, except for adults whose estimated rates were slightly below the AI level. Across lifestages, ADD values generally declined with age. Among the reported media, almost every lifestage followed a similar media ADD contribution pattern. Based on available data, oral ingestion from non-food sources were relatively low overall. However, the estimate for water ingestion may have been underestimated for young infants since ExpoFIRST v2.0 only included data on breastfeeding. In fact, previous studies reported that manganese intake rates from water in formula-fed young infants yielded the highest calculated dose compared to other lifestages [35]. This limitation of ExpoFIRST v2.0 may have led to underestimated manganese exposure rates in this lifestage. Instead, this illustrative case example predicted grains as the largest contributor to ADD across all lifestages. Consequently, the second row in Fig. 2 focuses specifically on ADD and LADD for grains, illustrating that ADD values are highest among infants and decline among subsequently older lifestages. Moreover, the contributions to manganese during adulthood to LADD were larger than other lifestage LADDs due to the longer duration of the adult lifestage.

#### Question 3: How has an environmental regulation affected aggregate exposure rates to a chemical across lifestages?

Endosulfan, an organochlorine insecticide, was selected to ask whether a policy change, in this case a US federal intervention, affected lifestage or media-specific aggregate oral exposure rates using ExpoKids. Endosulfan was commercially applied on crops starting in the 1950s [36]. US EPA assessed the endocrine disrupting chemical’s human health risks in 2002 and revised its human health and ecological risk assessments in 2007 [36]. Health assessments by international agencies have investigated the effects of endosulfan intake in food and water [37–39]. Exposure to endosulfan during prenatal and postnatal development can lead to neurodevelopmental effects [40]. Furthermore, high levels of endosulfan in the cord blood and breastmilk of pregnant women have been reported [41, 42]. Although data gaps exist in the understanding of specific critical windows of exposure, an animal study identified gestational and lactational exposure as sensitive developmental windows for endosulfan, leading to disruption of the nigrostriatal dopamine system development in male offspring [43]. As a result, historic endosulfan occurrence data can help explore the impacts of a regulatory decision that banned all uses of this pesticide in 2016 following a formal phase-out agreement in 2010 [36].

Dietary exposure to endosulfan before and after the 2010 phase-out was compared using data from the US Department of Agriculture’s (USDA) Pesticide Data Program (PDP) [44]. Specifically, trace detections of endosulfan sulfate (the primary metabolite of endosulfan degradation in soil and sediments) in food commodities were evaluated from PDP reports downloaded for all available years (1994–2016) [45]. No data outside of this timeframe were available at the time of data collection. Table 5 shows concentration values (positive detects only) for ExpoKids media sources tested for endosulfan sulfate residue for the years 1994–2010 and 2011–2016. PDP measurements were extracted to estimate mean endosulfan sulfate concentrations for six food groups (dairy, meat, fish, fruit, vegetables, and grains).

**Fig. 3:**
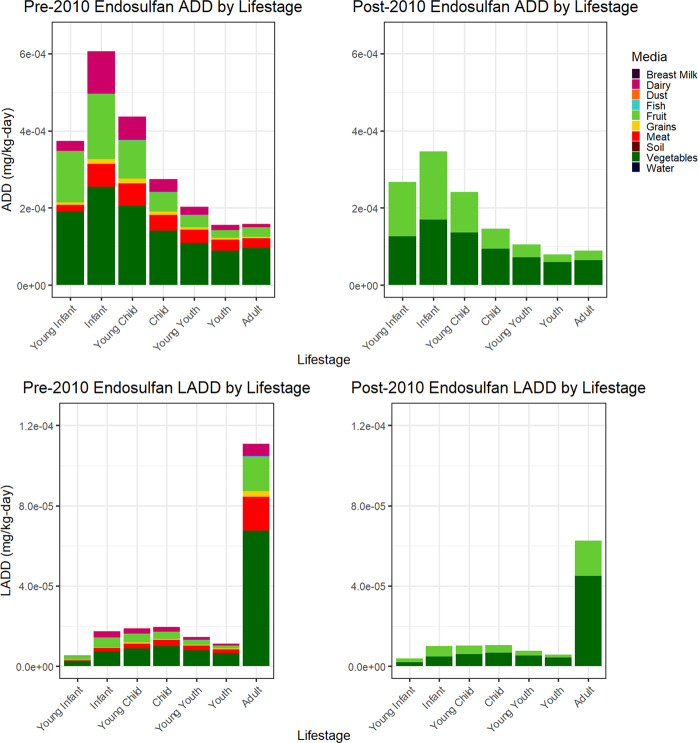
Endosulfan sulfate illustrative case example graphs: ADD and LADD by lifestage before the 2010 endosulfan US phase-out are shown in the left column and after the phase-out in the right column.

ExpoKids graphs were generated for both 1994–2010 and 2011–2016 to compare aggregate exposure before and after the 2010 start of the US endosulfan phase-out. Figure 3 shows aggregate ADD and aggregate LADD by lifestage ExpoKids graphs for dietary intake of endosulfan sulfate prior to 2010 (left column) and after 2010 (right column). For both time periods, infants experienced the largest ADD and the ADD estimates declined as age increased. In addition, graphs from both time periods showed that all childhood lifestages showed similar LADDs and that the adult LADD was highest. Based on PDP concentration data collected over these two time periods, endosulfan sulfate residues decreased to below analytical detection limits in all media except for fruits and vegetables for all lifestages after 2010. This would be expected since the crops whose last endosulfan usage dates occurred after 2010 were largely fruits and vegetables [36].

## Discussion

ExpoKids integrates information for a single route of exposure (oral) from multiple media across lifestages to visually display lifetime estimated aggregate chemical exposures for childhood and adult lifestages. In exploring publicly available tools, ExpoKids is the only identified exposure assessment tool that depicts aggregate exposure to any chemical for multiple lifestages using this set of illustrative comparative graphs. However, EPA’s exposure toolbox (ExpoBox; https://www.epa.gov/expobox) lists a variety of other tools that have also been developed for modeling aggregate exposure. Other proprietary tools may exist but are not publicly available. While these tools are powerful for estimating various exposures with a variety of inputs and specificity, each is built for a particular purpose that lacks the flexible data visualization of ExpoKids. For instance, SHEDS focuses on human activity patterns for chemical concentration and gathers exposure data from EPA field studies and published literature, IEUBK primarily estimates blood lead levels, and CEM was developed for consumer exposure scenarios [46–48]. These tools primarily provide calculated table outputs and lack the data visualization outputs that ExpoKids provides.

Since ExpoKids is coordinated with ExpoFIRST outputs, ExpoKids has the advantage of utilizing the recommended EFH lifestage-specific exposure factors. It can illustrate lifestage-specific aggregate exposure estimates for any chemical with media-specific exposure information. The ExpoKids v1.0 code (S-2) was developed in R, a publicly available, open-source software, for transparency. The user guide and code are available in the Supplementary Information (S-1 and S-2) and future updates to the R tool can be found on the EPA’s ExpoKids webpage (www.epa.gov/expobox/expokids-data-visualization-tool-aggregate-exposure-lifestage-and-media). Although these case examples were estimated using ExpoFIRST v2.0, an updated ExpoFIRST v2.1 was released in June 2019. ExpoKids was developed with ExpoFIRST ADD calculations in mind, but runs separately. Therefore, as ExpoFIRST revisions reflect EFH updates, ExpoKids can continue to display ADDs calculated with the most recent exposure factors. ExpoKids also has the flexibility to visually display exposure estimates for multiple scenarios, as exemplified in the three illustrative case examples. The DEHP case example highlighted individual media contributions to aggregate exposure across developmental lifestages, revealing both specific behaviors and dietary patterns that could impact lifestage aggregate exposures. The manganese case example showed the lifestage- and medium-specific contributions of these exposures relative to established health benefit and toxicity levels. The endosulfan case example depicted decreased media concentrations and lifestage exposure rates following the US phase-out and ban of endosulfan. Yet these illustrative case examples addressed only a fraction of the range of questions that could be addressed using ExpoKids.

Scoping decisions simplified data acquisition and input for the illustrative case examples. For instance, ExpoKids combined the smallest age groups from ExpoFIRST into the young infant and infant lifestages, resulting in a loss of specific information for newborn ADD. Since sample size was not a selection criterion, some studies with small sample sizes (*n* < 30) were included. Moreover, per capita central tendency estimates did not capture population variability; actual aggregate exposure likely varied across different populations. For example, ADDs estimated for groups with specific characteristics (e.g., families living near a contaminated area) may be higher compared to the general public. The ADD estimation also assumed that the media concentrations remained constant over time for all lifestages. This fixed chemical concentration captured only a snapshot of the population’s exposure at one point in time; to look at exposure as the population ages, temporal exposure concentration data would be needed. Similarly, the LADD was estimated separately for each lifestage and therefore, averaged the exposure for each lifestage over a lifetime. This limitation, inherent to the LADD equation, resulted in LADDs that did not account for ingestion during other lifestages. Therefore, total lifetime exposure may have been underestimated. Due to the limited availability of sex-specific data, differences in exposure by sex were not evaluated. Similarly, ExpoKids graphs did not represent comprehensive aggregate exposure from all routes and media for a given chemical due to a lack of chemical-specific data and the limited number of included media groups. Even though not all exposures were captured in ExpoKids, knowledge on the oral route of exposure was still gained.

ExpoKids also shared limitations with ExpoFIRST. For instance, point estimates were calculated rather than probabilistic distributions. ExpoFIRST does not use physiologically based pharmacokinetic (PBPK) models, so internal doses were not estimated. ExpoFIRST also does not evaluate the prenatal lifestage, although gestation may be a critical window of exposure; current data gaps render some exposure estimates during the gestational lifestage uncertain. In addition, the effectiveness of ExpoKids relies on the chemical concentration data that users input into ExpoFIRST and the exposure parameters selected. ExpoFIRST v2.0 did not have an option to evaluate infant formula consumption even though supplementing breastmilk with infant formula is commonly practiced in the USA; studies have indicated that ~24% of 12-month-olds breastfeed [49]. As a result, ExpoFIRST v2.0 overestimated breastmilk consumption and did not represent formula-fed infants who would have increased water consumption. After the development of ExpoKids, EPA updated the EFH (Chapter 3; https://cfpub.epa.gov/ncea/risk/recordisplay.cfm?deid=343661) to include data on drinking water intake among formula-fed infants to address this data gap. Therefore, ADD calculations made with these and future data updates may be used to calculate ADDs for visualization in ExpoKids.

### Challenges and opportunities

ExpoKids contributes to an improved understanding of relative aggregate exposure by lifestage for various oral media. ExpoKids can facilitate the investigation of a broad range of questions beyond those covered by the illustrative case examples, e.g., evaluating whether multiple chemicals with a similar mode of action reach a level of concern when the chemicals individually may not. For environmental chemical decisions requiring region-specific chemical concentration information, ExpoKids can be used by scientists to assess the aggregate risk among different geographic areas. Specifically, it could be asked: Do aggregate exposures differ for different settings (e.g., urban vs. rural)? Do they correlate with non-environmental factors (sex, behavior, regulation, etc.)? ExpoFIRST and ExpoKids could be used to explore these and other risk assessment questions for available exposure data from limited media for the oral route of exposure.

Some limitations of ExpoKids reflected data constraints. More publicly available data for chemical-specific media concentrations are needed. At this time, chemical monitoring can vary by media; however, comprehensive contaminant data for all media sources are needed to improve aggregate exposure modeling. For many developmentally toxic chemicals, the most sensitive critical window of exposure is expected to occur during prenatal development. Unfortunately, most chemicals lack a PBPK model for gestation due to data gaps. Yet some chemicals, including those presented in these illustrative case examples, have prenatal and postnatal critical windows. Therefore, developing methods to incorporate gestational exposure is crucial. In addition, data collected from multiple compatible data sources or from meta-analyses would offer a more comprehensive aggregate exposure assessment and allow for customized analyses in the future, depending on the availability of user-defined data identifying specific factors (i.e., sex, age, etc.).

By combining the functionality of ExpoFIRST with the visual graphic capabilities of R, ExpoKids facilitates comparisons of aggregate exposure by media both within and across lifestages. A broad range of ExpoKids users is envisioned. These include chemical risk assessors and managers running ExpoKids as a scoping tool to identify relevant exposure questions, as well as teachers using ExpoKids as an aggregate exposure teaching tool. Other potential users, such as pediatric health experts, may wish to use ExpoKids to explore differences in consumption patterns and exposure factors between and within children’s lifestages. Consequently, potential users can focus on relative contributions from different media to estimate the impacts of possible mitigation technologies or policies. Future efforts include enhancing public availability of this tool by converting ExpoKids into a web-based R Shiny application. As more exposure data for environmental chemicals become available, the capabilities of ExpoKids may be expanded.

## Supplementary information

Supplementary Information 1: ExpoKids Version 1.0 User Guide

Supplementary Information 2: ExpoKids Version 1.0 Code

Supplementary Information 3: Supplemental Tables

## Data Availability

The ExpoKids v1.0 user guide (S-1) and R code (S-2) are provided in the Supplementary Information.
