# Supplementary material for: ExpoKids: An R-based tool for characterizing aggregate chemical exposure during childhood
Source: J Expo Sci Environ Epidemiol. 2020 Oct 5;31(2):233–47. doi: 10.1038/s41370-020-00265-6 (PMC7952264; doi:10.1038/s41370-020-00265-6)
Supplement: Supplementary file 1 — Supplementary Information 1: ExpoKids Version 1.0 User Guide [file 41370_2020_265_MOESM1_ESM.docx]

# Supplementary Information 1: ExpoKids Version 1.0 User Guide

This file contains instructions on how to use ExpoKids.

## **Objective**

The goal of [ExpoKids Version 1.0 (https://www.epa.gov/expobox/expokids-data-visualization-tool-aggregate-exposure-lifestage-and-media)](https://www.epa.gov/expobox/expokids-data-visualization-tool-aggregate-exposure-lifestage-and-media) is to visually display aggregate exposure estimates representing relative exposure sources for a set of oral media, within and across lifestages. ExpoKids can be used as a standalone data visualization tool or by interfacing with the publicly available US EPA [Exposure Factors Interactive Resource for Scenarios Tool (ExpoFIRST; https://cfpub.epa.gov/ncea/efp/recordisplay.cfm?deid=344928).](https://cfpub.epa.gov/ncea/efp/recordisplay.cfm?deid=344928%20)^1^ The scope of the current version of the ExpoKids data visualization tool is limited to illustrating average daily dose (ADD) and lifetime average daily dose (LADD) from the oral exposure route for postnatal childhood lifestages (from birth to puberty) and includes adults as a comparator group. ExpoKids’ lifestage categories (Table S1) are based on the [Exposure Factors Handbook (EFH; https://cfpub.epa.gov/ncea/risk/recordisplay.cfm?deid=236252)](https://cfpub.epa.gov/ncea/risk/recordisplay.cfm?deid=236252).^2^ ExpoFIRST similarly relies on the EFH and user-defined media exposure concentration data to calculate ADDs.

Table S1: ExpoKids lifestages.

|  | **ExpoKids Lifestage** | **Age** | **Total Years  in Lifestage** |
| --- | --- | --- | --- |
| Childhood | Young Infant | 0 to < 1 year | 1 |
|  | Infant | 1 to < 3 years | 2 |
|  | Young Child | 3 to < 6 years | 3 |
|  | Child | 6 to < 11 years | 5 |
|  | Young Youth | 11 to < 16 years | 5 |
|  | Youth | 16 to < 21 years | 5 |
|  | Adult | 21 to < 70 years | 49 |
|  | Lifetime | Birth to < 70 years | 70 |

## **Step by Step Instructions**

As illustrated in Figure S1, there are five major steps to run ExpoKids: (1) Identifying oral exposure data; (2) Calculating ADD values; (3) Organizing ADD values in Excel; (4) Downloading ExpoKids in R; and (5) Running ExpoKids to generate graphs.

Figure S1. ExpoKids workflow from data input to graphical outputs of aggregate exposure by lifestage in ExpoKids: Step 1, identify exposure data for the 10 oral media included in ExpoFIRST. Exposure data can be identified via a number of sources, including the PubMed example provided or data collected by a researcher. Step 2, calculate the lifestage specific ADDs for these oral media data within ExpoFIRST or via another method. Step 3, organize the ADDs in an Excel file (see Table S2 template). Step 4, run the ExpoKids tool to generate graphs (see Table S4).


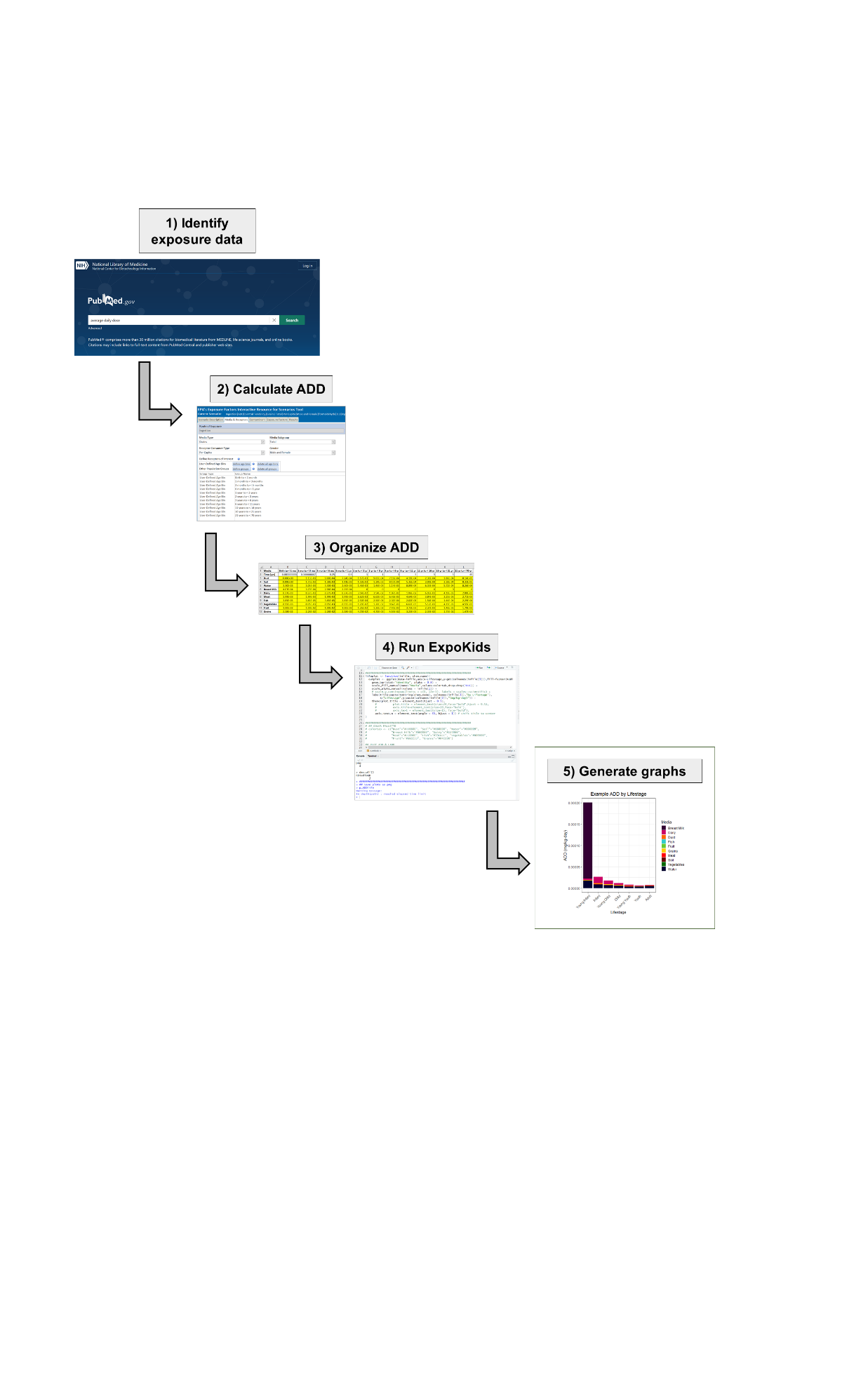


**Step 1:** Identify oral exposure data.
Users should identify the available and appropriate exposure data, either in the published literature or from original exposure studies, to a chemical or mixture of interest. The exposure study data quality is determined by the user’s criteria. By default, ExpoKids includes the following 10 oral media:

| - Breast Milk |
| --- |
| - Dairy |
| - Dust |
| - Fish |
| - Fruit |
| - Grains |
| - Meat |
| - Soil |
| - Vegetables |
| - Water |

Users may also collect data for additional oral media exposure of interest to be calculated alongside these default categories (see Step 2).

**Step 2:** Calculate ADD values by lifestage.
After identifying and collecting appropriate data sources on chemical concentrations in their oral exposure sources of interest, users calculate ADD values for each media exposure of interest and lifestage. ExpoKids was designed to generate graphs following ADD calculations using EPA’s ExpoFIRST. Users are encouraged to input chemical concentrations into ExpoFIRST because of the lifestage bin compatibility between ExpoKids and ExpoFIRST.^1^ If using ExpoFIRST, users can calculate ADDs by lifestage at [https://cfpub.epa.gov/ncea/efp/recordisplay.cfm?deid=344928](https://cfpub.epa.gov/ncea/efp/recordisplay.cfm?deid=344928%20) and follow the tool’s guide to run the desired exposure scenarios.^1^ If not using ExpoFIRST, users should double check that their ADD calculations are compatible with the lifestages defined in ExpoKids (see Table S1) and that appropriate exposure factors are used. Even if users do not choose to use ExpoFIRST, it is recommended that users consider applying EFH exposure factors, which can be found at <https://cfpub.epa.gov/ncea/risk/recordisplay.cfm?deid=236252>.^2^

**Step 3:** Organize ADD values in Excel table.
Once ADD values are calculated, users can save these values into the yellow highlighted section of the template table (Table S2) and save the file with the corresponding chemical or mixture name. Users may add rows for media types as needed if the default media groups are not relevant to their scenario. Alternatively, users may test ExpoKids with the provided sample table (Table S3). Both tables may be copy and pasted into Microsoft Excel to then be loaded by the ExpoKids R code (Step 4).

Table S2. Template ADD data table for user to fill in and upload to ExpoKids.

| **Media** | **Birth to < 1 mo** | **1 mo to < 3 mo** | **3 mo to < 6 mo** | **6 mo to < 1 yr** | **1 yr to < 2 yr** | **2 yr to < 3 yr** | **3 yr to < 6 yr** | **6 yr to < 11 yr** | **11 yr to < 16 yr** | **16 yr to < 21 yr** | **21 yr to < 70 yr** |
| --- | --- | --- | --- | --- | --- | --- | --- | --- | --- | --- | --- |
| **Time (yrs)** | 0.08333 | 0.16667 | 0.25 | 0.5 | 1 | 1 | 3 | 5 | 5 | 5 | 49 |
| **Dust** |  |  |  |  |  |  |  |  |  |  |  |
| **Soil** |  |  |  |  |  |  |  |  |  |  |  |
| **Water** |  |  |  |  |  |  |  |  |  |  |  |
| **Breast Milk** |  |  |  |  |  |  |  |  |  |  |  |
| **Dairy** |  |  |  |  |  |  |  |  |  |  |  |
| **Meat** |  |  |  |  |  |  |  |  |  |  |  |
| **Fish** |  |  |  |  |  |  |  |  |  |  |  |
| **Vegetables** |  |  |  |  |  |  |  |  |  |  |  |
| **Fruit** |  |  |  |  |  |  |  |  |  |  |  |
| **Grains** |  |  |  |  |  |  |  |  |  |  |  |

Table S3. Sample ADD data table ready to be uploaded into ExpoKids.

| **Media** | **Birth to < 1 mo** | **1 mo to < 3 mo** | **3 mo to < 6 mo** | **6 mo to < 1 yr** | **1 yr to < 2 yr** | **2 yr to < 3 yr** | **3 yr to < 6 yr** | **6 yr to < 11 yr** | **11 yr to < 16 yr** | **16 yr to < 21 yr** | **21 yr to < 70 yr** |
| --- | --- | --- | --- | --- | --- | --- | --- | --- | --- | --- | --- |
| **Time (yrs)** | 0.08333 | 0.16667 | 0.25 | 0.5 | 1 | 1 | 3 | 5 | 5 | 5 | 49 |
| **Dust** | 0.00E+0 | 4.94E-04 | 3.94E-04 | 3.17E-04 | 5.12E-04 | 4.23E-04 | 3.14E-04 | 1.83E-04 | 1.03E-04 | 8.15E-05 | 3.57E-05 |
| **Soil** | 0.00E+0 | 4.77E-05 | 3.81E-05 | 3.06E-05 | 4.12E-05 | 3.40E-05 | 2.52E-05 | 1.48E-05 | 8.26E-06 | 6.56E-06 | 2.29E-06 |
| **Water** | 1.33E-04 | 1.23E-04 | 1.33E-04 | 1.05E-04 | 5.89E-05 | 5.89E-05 | 4.61E-05 | 3.58E-05 | 2.56E-05 | 2.30E-05 | 3.33E-05 |
| **Breast Milk** | 1.06E-01 | 9.90E-02 | 7.78E-02 | 5.87E-02 | 0.00E+0 | 0.00E+0 | 0.00E+0 | 0.00E+0 | 0.00E+0 | 0.00E+0 | 0.00E+0 |
| **Dairy** | 1.28E-03 | 1.28E-03 | 1.28E-03 | 1.28E-03 | 5.46E-03 | 5.46E-03 | 3.04E-03 | 1.63E-03 | 1.07E-03 | 6.45E-04 | 4.32E-04 |
| **Meat** | 1.22E-04 | 1.22E-04 | 1.22E-04 | 1.22E-04 | 4.07E-04 | 4.07E-04 | 3.97E-04 | 2.85E-04 | 2.36E-04 | 2.00E-04 | 1.67E-04 |
| **Fish** | 1.27E-06 | 1.27E-06 | 1.27E-06 | 1.27E-06 | 8.24E-06 | 8.24E-06 | 7.61E-06 | 6.66E-06 | 5.14E-06 | 4.76E-06 | 7.55E-06 |
| **Vegetables** | 3.10E-05 | 3.10E-05 | 3.10E-05 | 3.10E-05 | 4.15E-05 | 4.15E-05 | 3.35E-05 | 2.29E-05 | 1.77E-05 | 1.45E-05 | 1.58E-05 |
| **Fruit** | 3.84E-05 | 3.84E-05 | 3.84E-05 | 3.84E-05 | 4.84E-05 | 4.84E-05 | 2.85E-05 | 1.43E-05 | 9.05E-06 | 5.58E-06 | 6.85E-06 |
| **Grains** | 1.91E-04 | 1.91E-04 | 1.91E-04 | 1.91E-04 | 3.94E-04 | 3.94E-04 | 3.82E-04 | 2.71E-04 | 1.97E-04 | 1.45E-04 | 1.23E-04 |

The Excel table file must contain ADD values for the following lifestages:

| - Birth to < 1 month |
| --- |
| - 1 month to < 3 months |
| - 3 months to < 6 months |
| - 6 months to < 1 year |
| - 1 year to < 2 years |
| - 2 years to < 3 years |
| - 3 years to < 6 years |
| - 6 years to < 11 years |
| - 11 years to < 16 years |
| - 16 years to < 21 years |
| - 21 years to < 70 years |

To be compatible with ExpoKids, all data tables must be in the same format as Tables S2 and S3. In other words, all age bins must be listed as columns and exposure media as rows. Additional exposure media rows may be added (or existing rows removed) depending on the users’ media of interest. The second row must correspond to the time duration in years of each age group and should not be changed from the template file. If desired, users can add or change the media of interest in their own table. Otherwise, users need only change the cells containing the ADD values (highlighted in yellow in Tables S2 and S3). The data table should be saved as a Microsoft Excel “.xls” or “.xlsx” file.

**Step 4:** Download the ExpoKids code.
To run ExpoKids, users must first [download R (https://www.r-project.org/)](https://www.r-project.org/), a free software environment for statistical computing and graphics. Download (or copy and paste) the ExpoKids v1.0 code (S-2) and open it in R.

**Step 5:** Generate ExpoKids graphs.
The only section of the code that the user needs to modify is outlined in the “User Inputs” section of the ExpoKids v1.0 code and appears as follows:

#############################################################################

## User Input Section

# ============================================================

# Required:

# -----------

# Path to Excel spreadsheet on your computer.

chempath <- "insert_path_here/chemname.xlsx"

# Chemical name

chemname <- "chemical_name"

# ============================================================

# Optional: - To run, uncomment saved variables below and adjust accordingly.

# -----------

# Adjust y-axis scale

# y_lims <- c(0, 1e-3)

#############################################################################

Only two variable names need to be changed in the R code; specifically, these variables define the path to the location of the ADD Excel table created in Step 3 on the user’s computer (“chempath”) and the name of the chemical or mixture of interest (“chemname”). An optional code chunk is also available to adjust the scale of the y-axis on the graphs (“y_lims”) which is not set to run (is commented out) in the default version of the code. If the user wishes to change the scale of the y-axis, this section will need to be uncommented (delete the “#” symbol in front of “y_lims”).

Once the User Inputs section has been edited, users can click the “Run” button
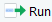
 to load the data table created in Step 3 and generate the graphs and tables of interest. The code will automatically produce all ExpoKids graphs and the user can view each by typing in the variable name (specified when saved) for the graphs and tables (see Tables S4 and S5). Moreover, ExpoKids will automatically save the tables as comma separated value (.csv) files and the graphs as portable network graphics (.png) files. The user can save additional file types by changing “png” in the “dev_copy” line in the R code to the desired file type. Additional customizations to the graphs and downloadable file types are available for R coders who wish to edit the ExpoKids code. For users interested in further R information or training, consider reading Garrett Grolemund and Hadley Wickham’s [*R for Data Science* (https://r4ds.had.co.nz/)](https://r4ds.had.co.nz/).^3^

Table S4 lists the graph types and explanations, graph variable names in the R code, and corresponding example graphs. Table S5 lists the generated tables and table variable names for ADD and lifetime average daily dose (LADD).

## **Outputs**

Table S4: ExpoKids graph outputs.

| **Graph Type** | **Variable Name** | **Example Graph** |
| --- | --- | --- |
| **1. ADD by lifestage:** Compares lifestages and media as relative contributors to an individual’s lifetime exposure. | p_ADDlife | 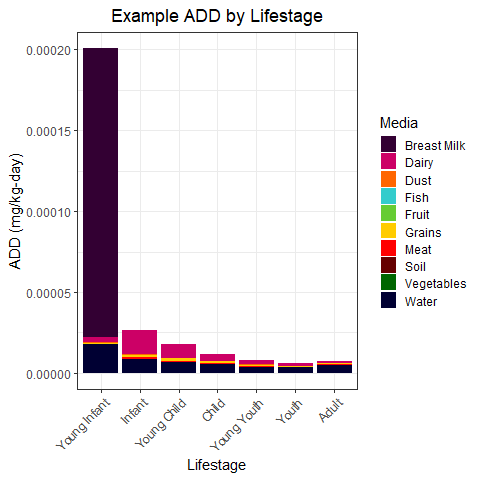 |
| **2. LADD by lifestage:** Compares lifestage and media ADD contributions scaled by the number of years an individual spends in each lifestage (time duration within a lifestage is considered). | p_LADDlife | 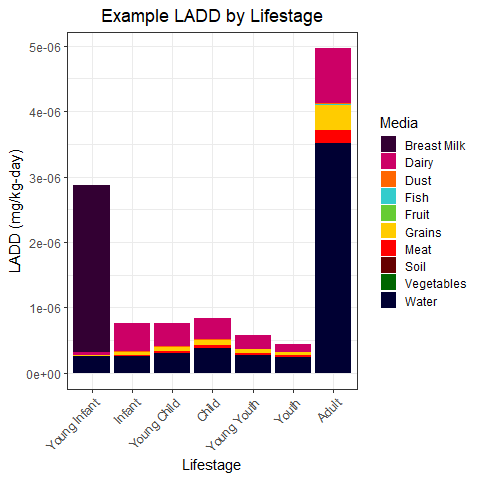 |

| **Graph Type** | **Variable Name** | **Example Graph** |
| --- | --- | --- |
| **3. Percent ADD by lifestage:** Calculates relative ADD contributions within a lifestage to relative percentages within a lifestage. | p_perlife | 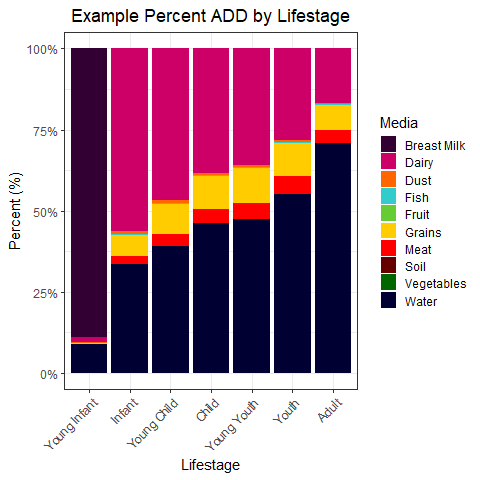 |
| **4. ADD by lifestage for an individual exposure pathway:** Focuses on illustrating the relative contribution between lifestages for a specific medium of interest (10 total). | p_ADD_*Media*, including:   - p_ADD_Breast Milk - p_ADD_Dairy - p_ADD_Dust - p_ADD_Fish - p_ADD_Fruit - p_ADD_Grains - p_ADD_Meat - p_ADD_Soil - p_ADD_Vegetables - p_ADD_Water | 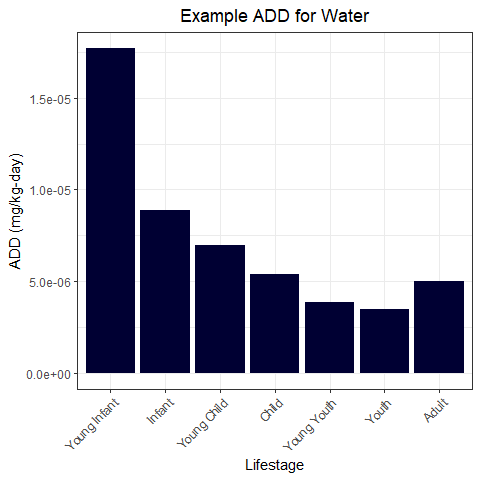 |

| **Graph Type** | **Variable Name** | **Example Graph** |
| --- | --- | --- |
| **5. LADD by lifestage for an individual exposure pathway:**  Focuses on illustrating the relative contribution between lifestages for a specific medium of interest (10 total) scaled by the number of years an individual spends in each lifestage. | p_LADD_*Media*, including:   - p_LADD_Breast Milk - p_LADD_Dairy - p_LADD_Dust - p_LADD_Fish - p_LADD_Fruit - p_LADD_Grains - p_LADD_Meat - p_LADD_Soil - p_LADD_Vegetables | 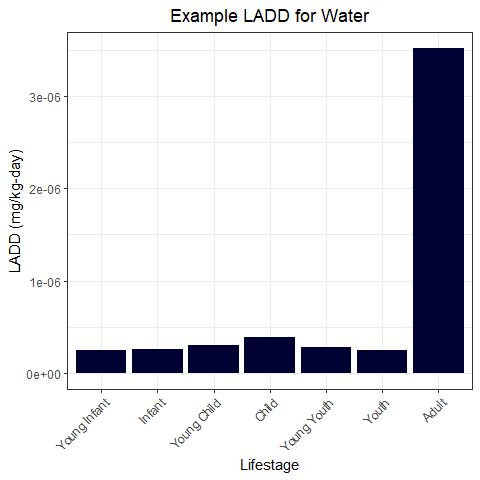 |

Table S5: ExpoKids table outputs.

| **Graph Type** | **Variable Name** | **Example Supplemental Table** |
| --- | --- | --- |
| **1. ADD Supplemental Table** | add_tab | 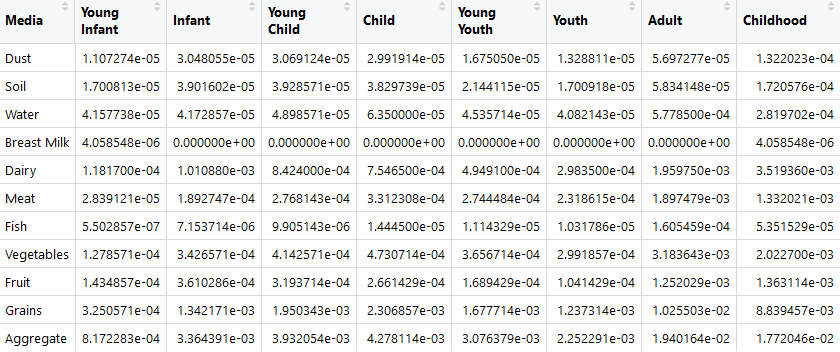 |
| **2. LADD Supplemental Table** | ladd_tab | 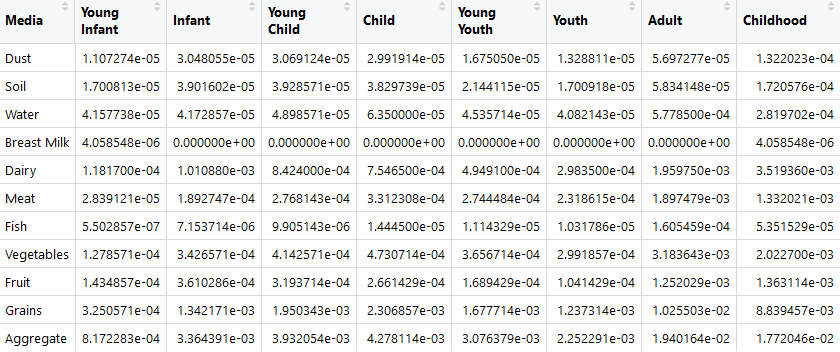 |

## **Assumptions and Limitations**

ExpoKids v1.0 presents non-R users with the ability to automatically generate and save aggregate exposure plots with limited coding knowledge. In addition, the ExpoKids data visualization tool does not allow the user to stray from the default lifestage age ranges. It is not fully integrated into one step with ExpoFIRST and therefore requires the user to perform the intermediary step of reorganizing the ADD data in Microsoft Excel before inputting these values into ExpoKids.

While ExpoKids was developed to work with ExpoFIRST, users are not required to use ExpoFIRST to calculate ADDs. If the ADDs are calculated correctly and formatted properly (see Table S2), users will be able to generate graphs in ExpoKids. The interpretation of ExpoKids graphs is highly dependent on the quality and appropriateness of the data input and ADD calculation assumptions for each lifestage and for each exposure medium. Users must be aware of how their ADD calculations are made. For example, ExpoFIRST v2.1 has a number of updates compared to ExpoFIRST v2.0 based on newly available exposure data that change the outcome of ADD calculations.^1^ Users should be aware of how such updates could affect their interpretations. Being aware of the scope of their question, users can combine the functionality of ExpoFIRST with the visual graphic capabilities of ExpoKids to compare aggregate exposure by media both within and across lifestages.

## **References**

1. Office of Research and Development (ORD). Exposure Factors Interactive Resource for Scenarios Tool (ExpoFIRST), Version 2.0 [Internet]. US Environmental Protection Agency; 2016. Available from: https://cfpub.epa.gov/ncea/risk/recordisplay.cfm?deid=322489

2. Exposure Factors Handbook: 2011 Edition [Internet]. Washington (DC); 2011 Sep. Report No.: EPA/600/R-090/052F. Available from: https://www.epa.gov/sites/production/files/2015-09/documents/techoverview_efh-complete.pdf

3. Wickham, H. and Grolemund, G., 2017. R for Data Science : Import, Tidy, Transform, Visualize, and Model Data. 1st Ed. Sebastopol, CA: O'Reilly Media, Inc.; 2017.

## **Figure Legend**

Figure S1. ExpoKids workflow from data input to graphical outputs of aggregate exposure by lifestage in ExpoKids: Step 1, identify exposure data for the 10 oral media included in ExpoFIRST. Exposure data can be identified via a number of sources, including the PubMed example provided or data collected by a researcher. Step 2, calculate the lifestage specific ADDs for these oral media data within ExpoFIRST or via another method. Step 3, organize the ADDs in an Excel file (see Table S2 template). Step 4, run the ExpoKids tool to generate graphs (see Table S4).
