# Supplementary material for: ExpoKids: An R-based tool for characterizing aggregate chemical exposure during childhood
Source: J Expo Sci Environ Epidemiol. 2020 Oct 5;31(2):233–47. doi: 10.1038/s41370-020-00265-6 (PMC7952264; doi:10.1038/s41370-020-00265-6)
Supplement: Supplementary file 2 — Supplementary Information 2: ExpoKids Version 1.0 Code [file 41370_2020_265_MOESM2_ESM.docx]

# Supplementary Information 2: ExpoKids Version 1.0 Code

This file contains the ExpoKids Version 1.0 code used to generate graphs for visualizing aggregate exposure for children compared to adults.

# ------------------------------

# title: "ExpoKids v1.0"

# author: "MD"

# date: "May 2020"

# output:

# png_imgs, pdf_document of ADD, LADD, and cumulative percent graphs

# purpose: This is the ExpoKids v1.0 code used to generate graphs for

# visualizing aggregate exposure for children compared to adults.

# ------------------------------

## Clear environment

remove(list = ls())

#####################################################################################################

## User Input Section

# ============================================================

# Required:

# -----------

# Path to Excel spreadsheet on your computer.

chempath <- "insert_path_here/chemname.xlsx"

# Chemical name

chemname <- "chemical_name"

# ============================================================

# Optional: - To run, uncomment saved variables below and adjust accordingly.

# -----------

# Adjust y-axis scale

# y_lims <- c(0, 1e-3)

#####################################################################################################

#####################################################################################################

### RUN ALL CODE BELOW THIS ###

#####################################################################################################

# ============================================================

# I. Prepare Environment.

# ------------------------------

# Install packages (if not already installed)

install.packages("tidyverse")

install.packages("reshape2")

install.packages("dplyr")

install.packages("scales")

devtools::install_github("tidyverse/readxl")

# Load packages

library(tidyverse)

library(reshape2)

library(dplyr)

library(readxl)

library(scales)

theme_set(theme_bw(base_size=15))

# ============================================================

# II. Load data

# ------------------------------

# A. Load Excel file.

datafile <- read_xlsx(chempath)

# ------------------------------

# B. Create lifestage data.

# Copy in data

chemfile <- data.frame(matrix(nrow=nrow(datafile),ncol=3))

chemfile[,1] <- datafile[,1]

# Calculate small infant lifestage (0-1 year)

for (i in 2:nrow(datafile)){

chemfile[i,2] <- ((datafile[1,2]*datafile[i,2])+

(datafile[1,3]*datafile[i,3])+

(datafile[1,4]*datafile[i,4])+

(datafile[1,5]*datafile[i,5]))/1 # small infant = 1 yr

}

# Calculate infant lifestage (1-3 years)

for (i in 1:nrow(datafile)){

chemfile[i,3] <- (datafile[1,6]*datafile[i,6]+

datafile[1,7]*datafile[i,7])/2

}

# Copy remaining lifestages

for (i in 8:ncol(datafile)){

chemfile[,i-4] <- datafile[,i]

}

## Rename column headers

chemfile <- chemfile[2:nrow(chemfile),]

names(chemfile) <- c("Media", "Young Infant", "Infant", "Young Child",

"Child", "Young Youth", "Youth", "Adult")

# ------------------------------

# C. Reorganize data files.

## Prepare file for graphing ADD

meltchemfile <- melt(chemfile,

id.vars="Media",

measure.var=c("Young Infant","Infant","Young Child","Child",

"Young Youth","Youth","Adult"),

na.rm=FALSE,

variable.name="Lifestage",value.name="ADD")

## Prepare file for graphing LADD

# Calculate time within each lifestage (tmeweight ADDs)

age <- 70 # assumed lifespan = 70 yrs

lifeyr <- c(0, 1/age, 2/age, 3/age, 5/age, 5/age, 5/age, 49/age)

# Create new weighted lifestages

wtchemfile <- chemfile[,1:8]

for (i in 2:ncol(wtchemfile)) {

wtchemfile[,i] <- Filter(is.numeric,(chemfile[,i]))*lifeyr[i]

}

mwt <- melt(wtchemfile,

id.vars="Media",

measure.var=c("Young Infant","Infant","Young Child","Child",

"Young Youth","Youth","Adult"),

na.rm=FALSE,

variable.name="Lifestage",value.name="LADD")

# ============================================================

# III. Plot graphs by media

# ------------------------------

# A. Prepare plotting function.

# Create color pallette for graphs

colortab <- c("Dust"="#FF6600",

"Soil"="#660000",

"Water"="#000033",

"Breast Milk"="#330033",

"Dairy"="#CC0066",

"Meat"="#FF0000",

"Fish"="#33CCCC",

"Vegetables"="#006600",

"Fruit"="#66CC33",

"Grains"="#FFCC00")

# Create function for plotting individual graphs by media

mediaplot <- function(infile, chem_name){

outplot <- ggplot(data = lifex, aes(x = Lifestage,y=get(colnames(infile[3])),

fill = factor(Lifestage))) +

geom_bar(stat = "identity", show.legend = FALSE, fill = colortab[i], alpha = 1) +

labs(title = paste(toString(chem_name), paste(colnames(infile[3]),"for",infile[i, 1])),

x = "Lifestage", y=paste(colnames(infile[3]),"(mg/kg-day)"))+

theme(plot.title = element_text(hjust = 0.5),

axis.text.x = element_text(angle = 45, hjust = 1))

# Adjust y-axis

if (exists('y_lims') == TRUE){

outplot <- outplot + scale_y_continuous(limits = y_lims, labels=scientific)}

return(outplot)

return(outplot)

}

# ------------------------------

# B. Create & save 10 ADD and LADD graphs by media.

# Create plotList

rm(plotList)

plotList <- list()

# Create & save 'By Media' ADD Plots

for (i in 1:nrow(chemfile)) {

# Create plots

lifex <- subset(meltchemfile,meltchemfile$Media==meltchemfile[i,1])

ADDmedia <- mediaplot(meltchemfile, chemname)

ADD <- paste("p_ADD",sep = "_", chemfile[i, 1])

assign(ADD, ADDmedia)

# Save plots

plotList[[i]] <- print(ADDmedia)

plotList[[i]]

dev.copy(png, filename=paste(chemname, chemfile[i, 1], "ADD_bylife.png", sep = '_'))

dev.off()

}

# Create & save 'By Media' LADD Plots

for (i in 1:nrow(chemfile)) {

# Create plots

lifex <- subset(mwt,mwt$Media==mwt[i,1])

LADDmedia <- mediaplot(mwt, chemname)

LADD <- paste("p_LADD", sep = "_", chemfile[i, 1])

assign(LADD, LADDmedia)

# Save plots

plotList[[i+nrow(chemfile)]] <- print(LADDmedia)

plotList[[i+nrow(chemfile)]]

dev.copy(png, filename=paste(chemname, chemfile[i, 1], "LADD_bylife.png", sep = '_'))

dev.off()

}

# ============================================================

# IV. Plot graphs by lifestages

# ------------------------------

# A. Prepare plotting function.

lifeplot <- function(infile, chem_name){

outplot <- ggplot(data=infile,

aes(x=Lifestage,

y=get(colnames(infile[3])),

fill=factor(Media))) +

geom_bar(stat="identity", alpha = 1) +

scale_fill_manual(name="Media",values=colortab,drop=drop(TRUE)) +

labs(title=paste(toString(chem_name), colnames(infile[3]),"by Lifestage"),

x="Lifestage",y=paste(colnames(infile[3]),"(mg/kg-day)")) +

theme(plot.title = element_text(hjust = 0.5),

axis.text.x = element_text(angle = 45, hjust = 1))

# Adjust y-axis

if (exists('y_lims') == TRUE){

outplot <- outplot + scale_y_continuous(limits = y_lims, labels=scientific)}

return(outplot)

return(outplot)

}

# ------------------------------

# B. Plot ADD & LADD graphs by lifestage.

p_ADDlife <- lifeplot(meltchemfile, chemname)

p_LADDlife <- lifeplot(mwt, chemname)

# ------------------------------

# C. Save plots as png

p_ADDlife

dev.copy(png, filename=paste(chemname,"ADD_bylife.png", sep = '_'))

dev.off()

p_LADDlife

dev.copy(png, filename=paste(chemname,"LADD_bylife.png", sep = '_'))

dev.off()

# ============================================================

# V. Plot graphs by cumulative percent.

# ------------------------------

# A. Prepare plotting function.

pctplot <- function(infile, chem_name){

outplot <- ggplot(data=infile,aes(x=Lifestage,y=get(colnames(infile[3])),

fill=factor(Media)))+

geom_bar(stat="identity",position="fill", alpha = 1) +

scale_y_continuous(labels=scales::percent) + # percent label axis

scale_fill_manual(name="Media",values=colortab,drop=drop(TRUE)) +

labs(title=paste(toString(chem_name), "Percent",colnames(infile[3]),"by Lifestage"),

x="Lifestage",y="Percent (%)")+

theme(plot.title = element_text(hjust = 0.5),

axis.text.x = element_text(angle = 45, hjust = 1))

return(outplot)

}

# ------------------------------

# B. Save cumulative percentage graph by lifestage as png.

# Plot

p_perlife <- pctplot(meltchemfile, chemname)

# Save plot

p_perlife

dev.copy(png, filename=paste(chemname,"Percent.png", sep = '_'))

dev.off()

# ============================================================

# VI. Create tables.

# ------------------------------

# A. Create new childhood & lifetime categories.

# Create childhood lifestage.

hood <- NA

for(i in 1:nrow(chemfile)){

hood[i]<-(((1*chemfile[i,2])+(2*chemfile[i,3])+(3*chemfile[i,4])+

(5*chemfile[i,5])+(5*chemfile[i,6])+(5*chemfile[i,7]))/21) # childhood = 21 yrs

}

# Create new table.

finchemfile <- chemfile

finchemfile$Childhood <- hood # Add childhood column

finchemfile$Lifetime<- rowSums(Filter(is.numeric,wtchemfile[,1:8])) # Add lifetime column

# Reorganize table.

mfin <- melt(finchemfile,

id.vars="Media",

measure.var=c("Childhood","Adult","Lifetime"),

na.rm=FALSE,

variable.name="Lifestage",value.name="ADD")

# ------------------------------

# B. Plot and save childhood, adult, lifetime graphs.

## Plot graphs

# Plot ADD graph

p_ADDfin <- lifeplot(mfin, chemname)

# Plot percent graph

p_perfin <- pctplot(mfin, chemname)

## Save plots

p_ADDfin

dev.copy(png, filename=paste(chemname,"Summary_ADD.png", sep = '_'))

dev.off()

p_perfin

dev.copy(png, filename=paste(chemname,"Summary_Percent.png", sep = '_'))

dev.off()

# ------------------------------

# C. Create finalized tables for display with childhood, lifetime, and aggregate columns.

## Create ADD table.

add_tab <- finchemfile

aggrow1<-colSums(Filter(is.numeric,add_tab))

# Add aggregate row to table.

add_tab[nrow(add_tab)+1,1]<-"Aggregate"

add_tab[nrow(add_tab),2:ncol(add_tab)]<-aggrow1

## Create LADD table.

ladd_tab <- wtchemfile

# Add childhood column.

ladd_tab$Childhood <- rowSums(Filter(is.numeric,wtchemfile[,1:7]))

#####################################################################################################

# ============================================================

# VII. Save and View outputs.

# ------------------------------

# A. View ADD & LADD tables.

## View ADD table

print('ADD Table')

add_tab

# Save table as CSV file

write.csv(add_tab, paste(chemname,"ADD_table.csv", sep = '_'))

# View LADD table

print('LADD Table')

ladd_tab

# Save table as CSV file

write.csv(ladd_tab, paste(chemname,"LADD_table.csv", sep = '_'))

# ------------------------------

# B. View all graphs.

# ADD by Media Plots

p_ADD_Dust

p_ADD_Soil

p_ADD_Water

p_ADD_BreastMilk

p_ADD_Dairy

p_ADD_Meat

p_ADD_Fish

p_ADD_Vegetables

p_ADD_Fruit

p_ADD_Grains

# LADD by Media Plots

p_LADD_Dust

p_LADD_Soil

p_LADD_Water

p_LADD_BreastMilk

p_LADD_Dairy

p_LADD_Meat

p_LADD_Fish

p_LADD_Vegetables

p_LADD_Fruit

p_LADD_Grains

# ADD by Lifestage Plot

p_ADDlife

# LADD by Lifestage Plot

p_LADDlife

# Cumulative Percentage Plot

p_perlife

# Childhood, Lifetime ADD Plot

p_ADDfin

# Childhood, Lifetime Percent Plot

p_perfin

# ------------------------------

# C. Save all plots to same PDF.

## Add to plotList

# By Life Plots

plotList[[length(plotList)+1]] <- lifeplot(meltchemfile, chemname)

plotList[[length(plotList)+1]] <- lifeplot(mwt, chemname)

# Remainder Plots

plotList[[length(plotList)+1]] <- pctplot(meltchemfile, chemname)

plotList[[length(plotList)+1]] <- lifeplot(mfin, chemname)

plotList[[length(plotList)+1]] <- pctplot(mfin, chemname)

## Create PDF

pdf(file=paste(chemname,"plots.pdf", sep = '_'))

plotList

dev.off()

############################################# END CODE #############################################
