# Supplementary material for: ExpoKids: An R-based tool for characterizing aggregate chemical exposure during childhood
Source: J Expo Sci Environ Epidemiol. 2020 Oct 5;31(2):233–47. doi: 10.1038/s41370-020-00265-6 (PMC7952264; doi:10.1038/s41370-020-00265-6)
Supplement: Supplementary file 3 — Supplementary Information 3: Supplemental Tables [file 41370_2020_265_MOESM3_ESM.docx]

# Supplementary Information 3: Supplemental Tables

This file contains supplementary data tables describing results of the search methods described in the main body of the paper that concern the studies selected for the chemical concentrations included in the three selected case examples.

**Tables S1.** PubMed search terms used to find concentration values in DEHP and manganese. The PubMed search was conducted in September 2018.

| **Search Term** | **Media** | **Number of Studies Returned** | **Selected Study** |
| --- | --- | --- | --- |
| Dust phthalate | Dust | 169 | Subedi et al., 2017 (16) |
| Soil phthalate detection | Soil | 46 | Lin et al., 2010 (17) |
| Drinking water phthalate | Water | 175 | Loraine et al., 2006 (18) |
| Breastmilk phthalate | Breastmilk | 61 | Hartle et al., 2018 (19) |
| Food phthalate detection | Dairy, meat, fish, vegetables, fruit, grains | 199 | Schecter et al., 2013 (20) |
| Dust manganese | Dust, soil | 336 | Gulson et al., 2014 (33) |
| Breastmilk manganese | Breastmilk | 128 | Klein et al., 2017 (34) |

**Tables S2.** Comparison of DEHP concentration values in breastmilk from included studies.

| **Study** | **Years Sampled** | **Chemical(s) Measured** | **Country** | **Number of Participants** | **Concentration [mg/mL]** | **Notes** |
| --- | --- | --- | --- | --- | --- | --- |
| Hartle et al., 2018 (1) | 2015 | MEHP, MEOHP, MEHHP | United States (California) | 21 | 7.07E-04 | Selected study for DEHP case example. |
| Del Bubba et al., 2018 (2) | N/A | DEHP | Italy | 9 | 3.50E-05 | Mean DEHP concentration reported. |
| Kim et al., 2015 (3) | 2012 | MEHP | South Korea | 62 | 2.08E-06 | Median MEHP concentration reported only. MEHHP & MEOHP were only detected in 1 sample. |
| Zimmerman et al., 2012 (4) | N/A | DEHP | Germany | 30 | 2.37E-06 | Median DEHP concentration reported. |
| Fromme et al., 2011 (5) | 2007-2008 | DEHP | Germany | 73 | 5.25E-06 | Mean DEHP Concentration reported. |
| Lin et al., 2011 (6) | 2001-2002 | MEHP, 5OH-MEHP, 5oxo-MEHP, 5cx-MEPP, 2cx-MEPP | Taiwan | 30 | 6.06E-07 | Mean of all metabolites reported. |
| [Latini et al., 2009](https://www.ncbi.nlm.nih.gov/pubmed/18684505) (7) | 2007 | MEHP | Italy | 62 | 8.40E-06 | Median MEHP concentration reported. |
| Hines et al., 2009 (8) | 2004-2005 | MEHP, MEOHP, MEHHP | United States (North Carolina) | 38 | 5.67E-07 | Mean MEHP concentration reported. Number of detections low for all metabolites. |
| Högberg et al., 2008 (9) | 2001 | DEHP, MEHP,  MEOHP, MEHHP | Sweden | 42 | 1.83E-05 | Mean of DEHP and MEHP concentrations reported. MEHHP & MEOHP not detected in any sample. |
| Zhu et al., 2006 (10) | 2003-2004 | DEHP | Canada | 86 | 2.29E-04 | Mean DEHP concentration reported. |
| Main et al., 2006 (11) | 1997-2001 | MEHP | Denmark | 65 | 9.50E-06 | Median MEHP concentration reported. |
| Main et al., 2006 (11) | 1997-2001 | MEHP | Finland | 65 | 1.30E-05 | Median MEHP concentration reported. |
| Mortensen et al., 2005 (12) | 1997-2001 | MEHP | Denmark | 36 | 1.30E-05 | Mean MEHP concentration reported. |
| Calafat et al., 2004 (13) | N/A | MEHP | United States (California) | N/A | 7.80E-06 | Mean MEHP concentration reported. |
|  | | | | **Average:** | **2.65E-05** |  |

**Tables S3.** Comparison of manganese concentrations in grains from included studies.

| **Source** | **Years Sampled** | **Media Tested** | **Country** | **Concentration [mg/g]** |
| --- | --- | --- | --- | --- |
| TDS, 2017 (36) | 2006-2013 | Grains | United States | 7.34E-03 |
| [Khan et al., 2013](https://www.sciencedirect.com/science/article/pii/S0278691513003189) (14) | 1993 | Maize, wheat, rice | Pakistan | 6.30E-01 |
|  |  |  | **Average:** | **3.19E-01** |

**Tables S4.** Comparison of endosulfan sulfate concentrations in vegetables from included studies.

| **Source** | **Years Sampled** | **Media Tested** | **Country** | **Concentration [mg/mL]** |
| --- | --- | --- | --- | --- |
| PDP Pre Phase-Out (15) | 1994-2010 | Vegetables | United States | 3.80E-05 |
| PDP Post Phase-Out (15) | 2011-2016 | Vegetables | United States | 2.53E-05 |
| Lozowicka et al., 2015 (16) | 2012-2014 | Cucumbers & tomatoes | Kazakhstan | 3.09E-06 |
| Fang et al., 2015 (17) | 2013 | Vegetables | China | < LOD |
| Chourasiya et al., 2014 (18) | 2012 | Vegetables | India | 1.93E-06 |
| TDS, 2006 (19) | 2003-2005 | Vegetables | United States | 3.64E-06 |
| Ntow, 2000 (20) | 2005 | Tomatoes | Ghana | < LOD |
| Lehotay et al., 2000 (21) | N/A | Apples, green beans,  & carrots | United States (Maryland) | 1.47E-04 |
|  |  |  | **Average:** | **3.90E-05** |

**Tables S5.** Estimated ADD [mg/kg-day] values for DEHP.

| **Media** | **Young Infant** | **Infant** | **Young Child** | **Child** | **Young Youth** | **Youth** | **Adult** |
| --- | --- | --- | --- | --- | --- | --- | --- |
| Dust | 3.40E-04 | 4.67E-04 | 3.14E-04 | 1.83E-04 | 1.03E-04 | 8.15E-05 | 3.57E-05 |
| Soil | 3.28E-05 | 3.76E-05 | 2.52E-05 | 1.48E-05 | 8.26E-06 | 6.56E-06 | 2.29E-06 |
| Water | 1.17E-04 | 5.89E-05 | 4.61E-05 | 3.58E-05 | 2.56E-05 | 2.30E-05 | 3.33E-05 |
| Breastmilk | 7.41E-02 | 0.00E+00 | 0.00E+00 | 0.00E+00 | 0.00E+00 | 0.00E+00 | 0.00E+00 |
| Dairy | 1.28E-03 | 5.46E-03 | 3.04E-03 | 1.63E-03 | 1.07E-03 | 6.45E-04 | 4.32E-04 |
| Meat | 1.22E-04 | 4.07E-04 | 3.97E-04 | 2.85E-04 | 2.36E-04 | 2.00E-04 | 1.67E-04 |
| Fish | 1.27E-06 | 8.24E-06 | 7.61E-06 | 6.66E-06 | 5.14E-06 | 4.76E-06 | 7.55E-06 |
| Vegetables | 3.10E-05 | 4.15E-05 | 3.35E-05 | 2.29E-05 | 1.77E-05 | 1.45E-05 | 1.58E-05 |
| Fruit | 3.84E-05 | 4.84E-05 | 2.85E-05 | 1.43E-05 | 9.05E-06 | 5.58E-06 | 6.85E-06 |
| Grains | 1.91E-04 | 3.94E-04 | 3.82E-04 | 2.71E-04 | 1.97E-04 | 1.45E-04 | 1.23E-04 |

**Tables S6.** Estimated ADD [mg/kg-day] values for manganese.

| **Media** | **Young Infant** | **Infant** | **Young Child** | **Child** | **Young Youth** | **Youth** | **Adult** |
| --- | --- | --- | --- | --- | --- | --- | --- |
| Dust | 7.75E-04 | 1.07E-03 | 7.16E-04 | 4.19E-04 | 2.35E-04 | 1.86E-04 | 8.14E-05 |
| Soil | 1.19E-03 | 1.37E-03 | 9.17E-04 | 5.36E-04 | 3.00E-04 | 2.38E-04 | 8.33E-05 |
| Water | 2.91E-03 | 1.46E-03 | 1.14E-03 | 8.89E-04 | 6.35E-04 | 5.72E-04 | 8.26E-04 |
| Breastmilk | 2.84E-04 | 0.00E+00 | 0.00E+00 | 0.00E+00 | 0.00E+00 | 0.00E+00 | 0.00E+00 |
| Dairy | 8.27E-03 | 3.54E-02 | 1.97E-02 | 1.06E-02 | 6.93E-03 | 4.18E-03 | 2.80E-03 |
| Meat | 1.99E-03 | 6.62E-03 | 6.46E-03 | 4.64E-03 | 3.84E-03 | 3.25E-03 | 2.71E-03 |
| Fish | 3.85E-05 | 2.50E-04 | 2.31E-04 | 2.02E-04 | 1.56E-04 | 1.44E-04 | 2.29E-04 |
| Vegetables | 8.95E-03 | 1.20E-02 | 9.67E-03 | 6.62E-03 | 5.12E-03 | 4.19E-03 | 4.55E-03 |
| Fruit | 1.00E-02 | 1.26E-02 | 7.45E-03 | 3.73E-03 | 2.37E-03 | 1.46E-03 | 1.79E-03 |
| Grains | 2.28E-02 | 4.70E-02 | 4.55E-02 | 3.23E-02 | 2.35E-02 | 1.73E-02 | 1.47E-02 |

**Tables S7.** Estimated ADD [mg/kg-day] values for endosulfan sulfate.

| **Pre Phase-Out** | | | | | | | | |
| --- | --- | --- | --- | --- | --- | --- | --- | --- |
| **Media** | **Young Infant** | **Infant** | **Young Child** | **Child** | **Young Youth** | **Youth** | **Adult** |  |
| Dairy | 2.58E-05 | 1.10E-04 | 6.12E-05 | 3.29E-05 | 2.16E-05 | 1.30E-05 | 8.72E-06 |  |
| Meat | 1.78E-05 | 5.92E-05 | 5.77E-05 | 4.14E-05 | 3.43E-05 | 2.90E-05 | 2.42E-05 |  |
| Fish | 1.58E-07 | 1.16E-06 | 9.99E-07 | 8.42E-07 | 6.52E-07 | 5.79E-07 | 8.96E-07 |  |
| Vegetables | 1.90E-04 | 2.55E-04 | 2.05E-04 | 1.41E-04 | 1.09E-04 | 8.89E-05 | 9.66E-05 |  |
| Fruit | 1.34E-04 | 1.68E-04 | 9.94E-05 | 4.97E-05 | 3.15E-05 | 1.94E-05 | 2.38E-05 |  |
| Grains | 6.20E-06 | 1.28E-05 | 1.24E-05 | 8.80E-06 | 6.40E-06 | 4.72E-06 | 3.99E-06 |  |
| **Post Phase-Out** | | | | | | | | |
| **Media** | **Small Infant** | **Infant** | **Small Child** | **Child** | **Small Youth** | **Youth** | **Adult** |  |
| Vegetables | 1.27E-04 | 1.70E-04 | 1.37E-04 | 9.36E-05 | 7.24E-05 | 5.92E-05 | 6.43E-05 |  |
| Fruit | 1.40E-04 | 1.76E-04 | 1.04E-04 | 5.20E-05 | 3.30E-05 | 2.03E-05 | 2.50E-05 |  |

# References

1. Hartle JC, Cohen RS, Sakamoto P, Barr DB, Carmichael SL. Chemical Contaminants in Raw and Pasteurized Human Milk. J Hum Lact. 2018 May;34(2):340–9.

2. Del Bubba M, Ancillotti C, Checchini L, Fibbi D, Rossini D, Ciofi L, et al. Determination of phthalate diesters and monoesters in human milk and infant formula by fat extraction, size-exclusion chromatography clean-up and gas chromatography-mass spectrometry detection. J Pharm Biomed Anal. 2018 Jan 30;148:6–16.

3. Kim S, Lee J, Park J, Kim H-J, Cho G, Kim G-H, et al. Concentrations of phthalate metabolites in breast milk in Korea: estimating exposure to phthalates and potential risks among breast-fed infants. Sci Total Environ. 2015 Mar 1;508:13–9.

4. Zimmermann S, Gruber L, Schlummer M, Smolic S, Fromme H. Determination of phthalic acid diesters in human milk at low ppb levels. Food Addit Contam Part Chem Anal Control Expo Risk Assess. 2012;29(11):1780–90.

5. Fromme H, Gruber L, Seckin E, Raab U, Zimmermann S, Kiranoglu M, et al. Phthalates and their metabolites in breast milk--results from the Bavarian Monitoring of Breast Milk (BAMBI). Environ Int. 2011 May;37(4):715–22.

6. Lin S, Ku H-Y, Su P-H, Chen J-W, Huang P-C, Angerer J, et al. Phthalate exposure in pregnant women and their children in central Taiwan. Chemosphere. 2011 Feb;82(7):947–55.

7. Latini G, Wittassek M, Del Vecchio A, Presta G, De Felice C, Angerer J. Lactational exposure to phthalates in Southern Italy. Environ Int. 2009 Feb;35(2):236–9.

8. Hines EP, Calafat AM, Silva MJ, Mendola P, Fenton SE. Concentrations of phthalate metabolites in milk, urine, saliva, and Serum of lactating North Carolina women. Environ Health Perspect. 2009 Jan;117(1):86–92.

9. Högberg J, Hanberg A, Berglund M, Skerfving S, Remberger M, Calafat AM, et al. Phthalate diesters and their metabolites in human breast milk, blood or serum, and urine as biomarkers of exposure in vulnerable populations. Environ Health Perspect. 2008 Mar;116(3):334–9.

10. Zhu J, Phillips SP, Feng Y-L, Yang X. Phthalate esters in human milk: concentration variations over a 6-month postpartum time. Environ Sci Technol. 2006 Sep 1;40(17):5276–81.

11. Main Katharina M., Mortensen Gerda K., Kaleva Marko M., Boisen Kirsten A., Damgaard Ida N., Chellakooty Marla, et al. Human Breast Milk Contamination with Phthalates and Alterations of Endogenous Reproductive Hormones in Infants Three Months of Age. Environ Health Perspect. 2006 Feb 1;114(2):270–6.

12. Mortensen GK, Main KM, Andersson A-M, Leffers H, Skakkebæk NE. Determination of phthalate monoesters in human milk, consumer milk, and infant formula by tandem mass spectrometry (LC–MS–MS). Anal Bioanal Chem. 2005 Jun 1;382(4):1084–92.

13. Calafat AM, Slakman AR, Silva MJ, Herbert AR, Needham LL. Automated solid phase extraction and quantitative analysis of human milk for 13 phthalate metabolites. J Chromatogr B. 2004 Jun 5;805(1):49–56.

14. Khan K, Lu Y, Khan H, Ishtiaq M, Khan S, Waqas M, et al. Heavy metals in agricultural soils and crops and their health risks in Swat District, northern Pakistan. Food Chem Toxicol. 2013 Aug 1;58:449–58.

15. Pesticide Data Program [Internet]. US Department of Agriculture (USDA); 2017. Available from: https://www.ams.usda.gov/datasets/pdp

16. Lozowicka B, Abzeitova E, Sagitov A, Kaczynski P, Toleubayev K, Li A. Studies of pesticide residues in tomatoes and cucumbers from Kazakhstan and the associated health risks. Environ Monit Assess. 2015 Sep 4;187(10):609.

17. Fang Y, Nie Z, Yang Y, Die Q, Liu F, He J, et al. Human health risk assessment of pesticide residues in market-sold vegetables and fish in a northern metropolis of China. Environ Sci Pollut Res Int. 2014 Nov 15;22.

18. Chourasiya S, Khillare PS, Jyethi DS. Health risk assessment of organochlorine pesticide exposure through dietary intake of vegetables grown in the periurban sites of Delhi, India. Environ Sci Pollut Res. 2015 Apr 1;22(8):5793–806.

19. Total Diet Study: Market Baskets 2004-1 through 2005-4 [Internet]. College Park, MD: US Food and Drug Administration (FDA); 2006. Available from: https://www.fda.gov/media/83172/download

20. Ntow W. Organochlorine Pesticides in Water, Sediment, Crops, and Human Fluids in a Farming Community in Ghana. Arch Environ Contam Toxicol. 2001 Jun 1;40:557–63.

21. SJ Lehotay. Analysis of pesticide residues in mixed fruit and vegetable extracts by direct sample introduction/gas chromatography/tandem mass spectrometry. J AOAC Int. 2000;83(3):680.
